# Dependence of the Concentration of Bioactive Compounds in *Origanum vulgare* on Chemical Properties of the Soil

**DOI:** 10.3390/plants10040750

**Published:** 2021-04-12

**Authors:** Asta Klimienė, Ramutis Klimas, Hanna Shutava, Liuda Razmuvienė

**Affiliations:** 1 Botanic Garden, Klaipėda University, H. Manto 84, 92294 Klaipėda, Lithuania; lrazmuviene@gmail.com; 2 Department of Medical Technologies, Faculty of Health Sciences, Klaipėda University, H. Manto 84, 92294 Klaipėda, Lithuania; klimas.ramutis@gmail.com; 3 Central Botanical Garden, National Academy of Sciences of Belarus, Surganova 2v, 220012 Minsk, Belarus; anna_shutova@mail.ru

**Keywords:** *O. vulgare*, total phenols, flavonoids, extractives, soil chemical properties, relationship

## Abstract

The aim of this study was to determine the dependence of the total phenolic, flavonoid, and extractive content in extracts of *Origanum vulgare* L. on the soil pH, humus, total nitrogen (N_total_), and plant-available/mobile phosphorus (P_2_O_5_), as well as potassium (K_2_O), total calcium (Ca), magnesium (Mg), and sodium (Na) concentrations. Experimental fields were formed in four locations in Lithuania. Soil and perennial *O. vulgare* samples were taken at three sites of each experimental field. A total of 12 samples of soil and 12 samples of the tested plant were collected and analyzed. The concentrations of bioactive compounds in plants are significantly (*p* < 0.05–0.01) affected by some chemical properties of the soil: the total phenolic content was reliably correlated with the soil pH, N_total_, Ca, Mg, and P_2_O_5_; the flavonoid content was correlated with the soil P_2_O_5_, K_2_O, Mg, and Na; and the extractive content was correlated with the soil humus, N_total_, and Na. The obtained results are useful for the development of the commercial cultivation of *O. vulgare*.

## 1. Introduction

Oregano (*Origanum vulgare*) is an aromatic, medicinal, and condiment herb belonging to the *Lamiaceae* family, commonly occurring throughout Asia, Europe, and northern Africa. In Lithuania, *O. vulgare* grows in the wild [1]. It is a perennial herb with a hairy stem up to 50 cm high and has oval leaves and purple flowers [2]. Recently, this plant has drawn more attention from consumers due to the antimicrobial, antifungal, insecticidal, and antioxidative effects of this herb on human health. However, oregano is most important as a culinary herb, used for its flavor around the world [2,3].

Extractives are a heterogeneous group of substances which can be extracted from plants. The oregano herb material contains mainly phenolic compounds as well as tannins, resins, flavonoids, bitter substances, sterols, and other compounds [4,5,6]. The dietary importance of medicinal plants is due to their phenolic phytochemicals which are a rich source of antioxidants [7,8]. These phenolic substances have advantageous outcomes on human health, in particular by preventing mutagenesis along with carcinogenesis [9,10,11]. Flavonoids and phenolic acids are the main types of phenolic compounds present in oregano. Flavonoid molecules are characterized by having at least one aromatic ring with one or more hydroxyl group attached [12]. Flavonoids are accumulated in plant tissues (such as leaves, flowers, stems, and roots) as a response to biotic and abiotic stress such as pathogen and insect attacks, UV radiation, and damage to the plant [13,14]. Moreover, phenolic compounds help plants survive and adapt to environmental disturbances through various physiological functions [15,16]. The total extractive substance refers to the percentage of material remaining after the removal of water. This indicator is more important for food materials, but for the content and composition of the essential oils of herbs, it is one of the most important characteristics.

The content of biological active compounds in plants depends on factors such as cultivar, geographical location, climate, environment conditions, harvesting time, and others [12,17,18,19].

Some of the soil chemical properties important for the growth of plants are the pH, humus, total nitrogen (N_total_), and plant-available/mobile phosphorus (P_2_O_5_) as well as potassium (K_2_O), calcium (Ca), magnesium (Mg), and sodium (Na). These components are not only important sources of materials for building up the structures of plant tissues, but are also actively involved in the metabolic activities within plants [20,21,22,23,24]. Other studies emphasize the importance of soil chemical composition for *O. vulgare* growth and the quality of their extracts, but these relationships are not indicated [25,26,27].

The aim of this study was to determine the dependence of total phenolic, flavonoid, and extractive content in extracts of *Origanum vulgare* L. on soil pH, humus, N_total_, P_2_O_5_, K_2_O, Ca, Mg, and Na concentrations.

## 2. Results and Discussion

### 2.1. Soil Chemical Characterization

The chemical properties of soils in the arable layer (0–20 cm) are given in Table 1.

The soil pH values in different experimental fields ranged between 5.75 and 7.80. According to the methodology of land productivity assessment in Lithuania, the soils vary from acidic to weakly alkaline [28]. The average pH value of the soils in all experimental fields (A–D) was 6.85. The acid/alkaline reaction of the soil solution is important for the mineralization of organic matter, solubility of the substances, intensity of microbiological processes [22], and for the growth of plants [20].

The amount of organic matter affects the potential fertility of the soil. A total of 80–85% of soil organic matter consists of humus [22]. Humus not only improves soil structure and the water and air regime, it also helps to store more heat and is an important source of nutrients for microorganisms and plants [21]. In the different experimental fields, humus varied from 3.34 to 10.7 %. According to the methodology of land productivity assessment in Lithuania [28], this soil quality indicator was quite high. 

According to the total nitrogen (N_total_), the arable layer of soils is divided into low nitrogen up to 0.2%, medium nitrogen up to 0.21–0.3%, adequate nitrogen up to 0.31–0.4%, and high nitrogen over 0.4% [29]. In separate fields, the soil N_total_ ranged from 0.13 (D) to 0.64% (C). Thus, in soils, the total nitrogen ranged from low to high. Nitrogen is needed for all plant growth processes. In the case of insufficient nitrogen content in the soil, the leaves of plants are small and light green, and begin to turn yellow, and the vegetation period of the plants is shortened [30].

The soil plant-available phosphorus (P_2_O_5_) in experimental fields varied from 112.70 (C) to 689.55 mg kg^−1^ (A). Thus, the phosphorus content in the soils was sufficient [28]. Phosphorus is an essential component in many vital plant processes. In the case of insufficient phosphorus in the soil, plants develop and grow poorly, and their leaves curl [31].

According to the plant-available potassium (K_2_O), the soils in our research were high quality. K_2_O ranged from 200.50 (C) to 1110.05 mg kg^−1^ (A). Potassium is important for ensuring optimal plant growth. It is an activator of photosynthesis, enzymes, and metabolism in plants [32]. 

The total content of calcium (Ca) in soils varied from 0.336 up to 2.05%, magnesium (Mg) from 0.239 up to 0.616%, and sodium (Na) ranged from 0.334 to 0.368%. Thus, the lowest content of Ca, Mg, and Na was in the soil of experimental field D, and the highest in experimental field B. According to Jakovljevic et al. [23], all mentioned alkali metals present in silicate rock-forming minerals and later are transferred into a mineral fraction of soils. Their content depends on the rate of mineral weathering and the rate of leaking of weathered products. The available forms of Ca, Mg, and Na are their exchangeable cations and various salts in the soil solution. These alkaline elements are important in plant nutrition. Ca is an essential component for root development, and Mg is important for photosynthesis. Na plays major beneficial roles in plant metabolism [33].

### 2.2. Contents of Bioactive Compounds in Plant Extracts

The concentrations of biologically active compounds in the *O. vulgare* raw materials of the extracts are presented in Table 2.

The total phenolic content in plants growing in the different experimental fields ranged from 2.98 to 3.69%. Therefore, in the investigated plants, a lower quantity of phenolic compounds was found in our study than in *O. vulgare* from countries with warmer climates [3,19,34,35]. This may also be due to the chemotypic features of the studied samples of *O. vulgare*, since the literature contains numerous data on the existence of several chemotypes differing in composition and amount of phenolic and terpene compounds in the essential oils of oregano [36,37,38]. In European populations of oregano, the chemotype of linalool predominates; there is also a cymyl chemotype or sabinyl chemotype that co-occurs with a sesquiterpene-type poor in monoterpenes [39]. Chemotypes with a relatively low content of phenolic compounds can also be valuable from a practical point of view, since they usually have a more pleasant floral aroma, which is associated with higher concentrations of linalool, and can be widely used in the cosmetic and perfume industry [40]. Phenolic compounds have antioxidant, antimicrobial, anticarcinogenic, and immunostimulant impacts not only for humans but also for animals [7].

Flavonoids make up the largest part of phenolic compounds [41]. The obtained amounts of flavonoids in the tested plant extracts ranged from 0.26 to 1.99%.

The content of extractives is the dry matter content of the extract obtained by extraction with a binary ethanol–water mixture per 100 g of plant material. The concentration of extractives can be considered one of the indicators of general biological activity, since the literature contains data on the best extraction of the most biologically active substances when using binary mixtures of extractants [42,43,44]. The content of extractive substances in plants of separate experimental fields varies from 14.13 to 18.12%. 

### 2.3. Relationship between Bioactive Compounds of Plants and Soil Chemical Parameters

The relationships between plant bioactive compounds and soil chemical parameters identified in our studies are given in Table 3.

The total phenolic content in *O. vulgare* was significantly positively correlated with the soil pH (r = 0.832), total nitrogen (r = 0.515), calcium (r = 0.842), and magnesium (r = 0.559), and negatively correlated with soil mobile phosphorus (r = −0.511).

The flavonoid content in tested plants is significantly affected by soil mobile phosphorus (r = 0.776), mobile potassium (r = 0.993), magnesium (r = 0.643), and sodium (r = −0.843).

The extractive content in plants was statistically significantly correlated with soil humus (r = 0.687), total nitrogen (r = 0.678), and sodium (r = −0.505).

The correlation between other plant bioactive compounds and soil chemical properties was not statistically significant. The data in Table 3 show that most of the correlations were positive (17 of 24).

In other similar research have analyzed only the relationship between soil nitrogen and oil yield, essential oil content in *O. vulgare*. Nitrogen significantly increased oil yield of Greek oregano (*Origanum vulgare* ssp. *hirtum* (Link) Ietswaart) [45]. A positive correlation coefficient between nitrogen and essential oil content in Egyption oregano (*Origanum syriacum* L. var. aegyptiacum Tackh) was determined. Nitrogen increased the biosynthesis of phenolic compounds such as thymol and carvacrol [46]. 

## 3. Materials and Methods

### 3.1. Study Area and Sampling

In May 2018, deep ploughing experimental fields were formed in four locations in Lithuania: A (in the Petronys village, Pakruojis district, northern Lithuania; 56°12′10.5″ N, 23°56′26.1″ E), B (in the Gintalai village, Telšiai district, northwestern Lithuania; 55°44′45.2″ N, 22°21′13.6″ E), C (in the Medsėdžiai village, Klaipėda district, western Lithuania; 55°39′26.7″ N, 21°28′53.2″ E), and D (in the Botanical Garden of Klaipėda University, Klaipėda city, western Lithuania; 55°45′02.5″ N, 21°08′02.5″ E). The experimental fields in different locations were 18 square meters in area (1 × 18 m^2^). Each experimental field in all locations consisted of black soil. The cultivation technology applied was also the same in all places. Oregano seedlings were planted in the first year. In June 2019 (dry season), the soil (in the arable layer of a 0–20 cm depth) and perennial *O. vulgare* samples were taken at three sites (beginning, middle, end) of each experimental field. A metal probe (drill) for soil sampling was using. Two to three plants were taken from one point. Three samples of soil and 3 samples of plants were collected from each experimental field. Each sample was placed in separate bags. Thus, a total of 12 samples of soil and 12 samples of the tested plants were collected. The collected plant material (stems, leaves and flowers) was air-dried in darkness at an ambient temperature. The dried plant material was cut up and stored in paper bags (*n* = 12) until needed (in September).

### 3.2. Agrochemical Analysis of Soils

All the samples of the soil were air-dried; the visible roots and plant residue were manually removed, and chemical analysis (pH, humus, N_total_, P_2_O_5_, K_2_O, Ca, Mg, and Na) was carried out at the Agrochemical Research Laboratory of the Institute of Agriculture, Lithuanian Research Centre for Agriculture and Forestry.

Soil pH was measured in a 1M KCl suspension according to the standard ISO 10390:2005 [47] (soil-solution ratio 1:2.5) using a pH-meter IONLAB. Humus was found by determination of organic carbon (C) by the Tyurin method modified by Nikitin [48] using a spectrophotometer Cary 50 (VARIAN). A correction factor of 1.724 was used to calculate humus from organic C. Soil total nitrogen (N_total_) was determined by the Kjeldahl method according to the standard ISO 11261:1995 [49], plant-available/mobile phosphorus (P_2_O_5_) as well as potassium (K_2_O) were determined by the Egner–Riehm–Domingo method [50]. The total calcium (Ca), magnesium (Mg), and sodium (Na) concentration in the soil was determined by using an atomic absorptium spectrophotometer (AAS) after the extraction with ammonium acetate [51].

### 3.3. Phytochemical Analysis of Plants

Extraction of all plant samples and the chemical analysis of bioactive compounds (total phenolic, flavonoid, and extractive content) was carried out at the Applied Biochemistry Laboratory of the Central Botanical Garden of the National Academy of Sciences of Belarus. 

The extraction of dried and ground plant samples was carried out in 2 stages using an 80% ethanol solution. A total of 2 g of plant material (accurately weighed) was placed in a conical flask, where 20 mL of 80% ethanol was added; the flask was closed with a stopper, weighed, and left alone for 1 h. Then, the flask was connected to a reflux condenser, heated to boiling in a water bath, and the liquid was kept at a slight boil for 30 min. The liquid was thoroughly shaken and filtered through a dry filter into a dry flask. The residue was re-poured with 20 mL of ethanol solution and boiled again for 30 min. It was filtered by transferring the rest of the plant material to the filter and by washing with portions of 80% ethanol. The filtrates were combined until reaching a total of 50 mL, and used for determination.

The total content of phenolic compounds in the dry matter of the extracts was determined using the modified Folin–Ciocalteu method [52].

The determination of flavonoids was carried out using the reaction of complexation with aluminum chloride [53]. To determine the total content of flavonoids, 2 mL of the analyzed solution was placed in a 25 mL volumetric flask; 1 mL of a 10% solution of aluminum chloride in ethanol was added until reaching the mark with 96% alcohol. After 30 min, the optical density of the solution was measured on an Agilent 8453 spectrophotometer at a wavelength of 410 nm. A mixture of 2 mL of a sample, 0.1 mL of concentrated acetic acid, and 96% ethanol to 25 mL was used as a reference solution. To construct the calibration line, rutin was used in the concentration range of 0.01 to 0.54 g/L.

To determine the extractives in the extracts, a known volume of the extract was placed in a weighing bottle, evaporated in a water bath, dried for 3 h at 102.5 °C, then cooled in a desiccator for 30 min and weighed.

### 3.4. Statistical Analysis

The measurement data were processed using the Statistica 10 StatSoft Inc. (Tulsa, OK, USA) program [54]. The soil and plant chemical results are presented as arithmetic means and standard errors (±SE). Pearson‘s correlation coefficients (linear relationships) between the bioactive compounds of *O. vulgare* and the parameters of the soil chemical assessment were calculated. Each determined coefficient (r) was then tested for statistical significance with Student‘s t-test for n-2 degrees of freedom. Correlation was considered statistically significant at *p* < 0.05.

## 4. Conclusions

The acidic soil reaction, a lower supply of humus, total nitrogen, mobile potassium, calcium, and magnesium against the background of a sufficiently high content of mobile phosphorus and sodium in the soil led to a lower content of total phenols and a lower content of extractives in the raw material of oregano. The flavonoid content in tested plants is significantly positively affected by soil mobile phosphorus, mobile potassium, magnesium, and significantly negatively affected by soil sodium.

In general, the results of this study demonstrate the high dependence of total phenolic, flavonoid, and extractive content in the extracts of *Origanum vulgare* L. on some chemical properties of the soil. The obtained results will be useful for the development of the commercial cultivation of *O. vulgare*.

## Figures and Tables

**Table 1:** Soil chemical properties (means ± SE).

Experimental Field	pH_KCl_	Humus, %	N_total,_ %	P_2_O_5,_ mg kg^−1^	K_2_ O_5_, mg kg^−1^	Ca, %	Mg, %	Na, %
A	7.10 ± 0.20	5.53 ± 0.15	0.235 ± 0.02	689.55 ± 231.75	1110.05 ± 121.45	1.379 ± 0.40	0.570 ± 0.11	0.246 ± 0.11
B	7.80 ± 0.00	3.34 ± 0.44	0.141 ± 0.02	192.25 ± 41.75	438.65 ± 97.85	2.050 ± 1.06	0.616 ± 0.19	0.368 ± 0.02
C	6.75 ± 0.05	10.70 ± 0.20	0.640 ± 0.05	112.70 ± 1.40	200.50 ± 19.30	1.220 ± 0.01	0.278 ± 0.01	0.337 ± 0.03
D	5.75 ± 0.05	3.94 ± 0.48	0.130 ± 0.01	561.55 ± 2.75	322.00 ± 11.10	0.336 ± 0.01	0.239 ± 0.01	0.334 ± 0.04
A-D	6.85 ± 0.75	5.88 ± 2.92	0.286 ± 0.21	389.01 ± 269.54	517.80 ± 360.85	1.246 ± 0.83	0.426 ± 0.20	0.321 ± 0.07

**Table 2:** Concentrations of bioactive compounds in the dry matter of *O. vulgare* (means ± SE).

Experimental Field	Total Phenolic Content, %	Flavonoid Content, %.	Extractive Content, %.
A	3.59 ± 0.13	1.99 ± 0.10	18.12 ± 1.92
B	3.64 ± 0.43	0.83 ± 0.06	15.90 ± 1.61
C	3.69 ± 0.23	0.26 ± 0.04	18.11 ± 1.53
D	2.98 ± 0.05	0.69 ± 0.06	14.13 ± 1.40
A–D	3.48 ± 0.29	0.94 ± 0.64	16.55 ± 1.68

**Table 3:** Correlation between bioactive compounds of *O. vulgare* and soil chemical parameters (in all experimental fields, A–D).

*O. vulgare*	Soil							
	pH	Humus	N_total_	P_2_O_5_	K_2_O	Ca	Mg	Na
Total phenolic content	0.832 **	0.461	0.515 *	−0.511 *	0.206	0.842 **	0.559 *	−0.074
Flavonoid content	0.265	−0.388	−0.449	0.776 **	0.993 **	0.189	0.643 *	−0.843 **
Extractive content	0.466	0.687 *	0.678*	−0.158	0.406	0.457	0.291	−0.505 *

Significant statistical correlation: * *p* < 0.05; ** *p* < 0.01.
